# The twisted path to sacredness: a grounded theory study of irrational religious orientation and its psycho-sociological implications

**DOI:** 10.1186/s40359-024-01858-8

**Published:** 2024-06-20

**Authors:** Ziang Wang, Yinglin Luo, Xuan Cao, Jindong Jiang

**Affiliations:** 1 Hangzhou Normal University, Hangzhou, Zhejiang China; 2 Hong Kong Ruyi Culture Limited, HongKong, China

**Keywords:** Generation Z, Irrational religious orientation, Grounded theory, Digital influence, Response strategies

## Abstract

This research delves into the nuances, origins, and societal effects of irrational religious orientations within China’s Generation Z, employing grounded theory methodology for a comprehensive analysis. The focus is on those born between 1995 and 2010, a demographic raised amidst rapid information technology growth and significantly influenced by digitalization and globalization. The study identifies three primary dimensions of irrational religious orientations in Generation Z: religious spiritual dependence, religious instrumental tendency, and religious uniqueness identity. These are shaped by factors such as the overwhelming influx of information via digital media, societal pressures and psychological dilemmas, conflicts in values and identity crises, as well as feelings of social isolation and the need for group belonging. To address these trends, the study suggests several interventions: enhancing multicultural and values education, implementing stricter online information regulation and literacy programs, boosting mental health awareness and support, and fostering engagement in social and cultural activities. These recommendations are essential for comprehensively understanding and effectively responding to the irrational religious orientations of Generation Z, ultimately contributing to their overall well-being and healthy development.

## Introduction

Generation Z’s deep engagement with technology significantly influences their values, lifestyles, and worldviews, including their religious inclinations [1–2]. Studies by Davis and Venkatesh et al. underscore their dependency on technology, driven by its perceived usefulness and ease of use [3–5]. This dependence is shaped by how well technology meets their needs, the effort involved, and its fit with their social milieu [4]. Their digital habits, including the use of social media, not only shape their health behaviors but also their religious attitudes, steering them towards pragmatic rather than traditional religious practices [2]. Understanding these digital behaviors is crucial for appreciating how they affect Generation Z’s lifestyle choices and religious perspectives [1–2, 4−5].

Regarding the “irrational religious orientation” in China’s Generation Z, it’s a multifaceted phenomenon influenced by various factors. Li Chen, Sheng Zeng, and Zaizhen Tian’s study challenges the notion of blind religious adherence among Generation Z, suggesting their religiosity is based on rational considerations of religious rewards [6]. Jurnal Pendidikan et al.‘s research implies that Generation Z’s openness to religion might indicate a moderate, flexible approach to beliefs [7]. This contradicts interpretations of their religious behavior as irrational. Demir’s study reveals Generation Z’s adoption of secular, transhumanist values like individuality and critical thinking, potentially influencing their religious orientations [8]. These findings highlight the need for future research to adopt a nuanced approach, considering the impacts of social change, globalization, and generational shifts on Generation Z’s religious orientations [6–8]. Such research could lead to strategies promoting balanced religious beliefs and practices in this demographic.

### Literature review

The exploration of religious orientation as a key influencer of human behavior, interpersonal relationships, and mental health has revealed a complex duality in its impacts, as evidenced by a range of studies. G. Allport and J. M. Ross’s seminal work highlights how religious orientation can significantly contribute to prejudice, linking certain prejudiced behaviors to specific religious orientations, notably those with indiscriminate favoritism towards religion [9].

Further, the interaction between religious orientation and mental health is intricate. M. Janbozorgi and F. Aliakbari, Dawood Taqvaei, and Z. Pirani, delve into the potential therapeutic aspects of religious orientation, suggesting a beneficial connection with mental health [10].

Religious orientation’s influence extends to media engagement, as Ahmad Saifalddin Abu-Alhaija et al. demonstrate its impact on viewer loyalty and perceptions in satellite TV consumption [11]. Similarly, a study links religious orientation with consumer purchasing behavior influenced by social media advertising, indicating an economic behavioral impact [12].

The realm of pro-social behavior, particularly among young women, is also explored, focusing on how religious beliefs shape charitable actions and empathy [13]. Conversely, some studies reveal potential negative implications of religious orientation, such as ‘extrinsic religious orientation’ correlating negatively with well-being [14], or influencing job-related stress levels [15].

Noteworthy contributions also include research on religious orientation’s relation to death anxiety in the elderly [16], depression in college students [17], and reproductive behaviors in women [18]. These studies collectively reaffirm the broad and significant impact of religious orientation on diverse life aspects.

In summary, the synthesis of religious orientation literature encompasses a vast array of domains, ranging from media consumption and mental health to societal and economic behaviors. The effects are varied and heavily dependent on individual experiences with religion, highlighting a multifaceted relationship between religious orientation and its influence on human life. This literature review emphasizes the need for more comprehensive and nuanced research to better understand these dynamics [9–19].

The existing literature on religious orientation predominantly focuses on Western contexts, underscoring a significant gap in research concerning non-Western societies, particularly China. Notably, the religious inclinations of Chinese youth, especially Generation Z, remain insufficiently explored. Chen, Zeng, and Tian found that religiosity among China’s Generation Z is notably higher than the national average, influenced by factors like practical benefits and religious socialization [20]. This underscores the importance of considering the unique cultural and social context in religious studies. Minkov and Kaasa’s study in Africa also highlights the often-neglected cultural differences in religion, sometimes misinterpreted as racial or ethnic disparities [21]. These insights call for a recontextualization of religious orientation research, particularly in non-Western settings, to enhance its relevance and accuracy.

In China, research on Generation Z’s religious inclinations predominantly focuses on rational factors that drive their religious choices [6], such as tangible benefits over supernatural elements. However, this perspective, aligning with theories from Stark and Finke, and Hartmut Rosa, largely omits the exploration of irrational or non-rational factors. This aligns with theories by Stark and Finke, and Hartmut Rosa [22], but largely ignores the role of irrational or non-rational factors [23]. Studies on religious moderation and the impact of Internet use on religious authority choices tend to focus on rational aspects [8]. In contrast, research on older populations reveals insights into irrational religious beliefs through acceptance and commitment therapy and the role of doubt in religious education, which could provide useful perspectives for studying Generation Z’s irrational beliefs [24–25].

In essence, while current literature provides critical insights, it largely overlooks the irrational elements of religious inclination in Generation Z. Exploring these aspects in future research could offer a more comprehensive understanding of this demographic’s religious dynamics.

### Purpose of the study

The research purpose of the study is to delve into the nuances, origins, and societal effects of irrational religious orientations within China’s Generation Z using grounded theory methodology. The study aims to provide a comprehensive analysis of these orientations, shaped by factors such as the influx of information via digital media, societal pressures, and psychological dilemmas. Additionally, it suggests several interventions to address these trends, ultimately contributing to the overall well-being and healthy development of this demographic.

### Methodology

The researcher’s analysis utilizes grounded theory, a methodology developed by Glaser and Strauss, which focuses on deriving theories from data rather than adhering to a pre-existing framework [26–28]. This approach is particularly effective in social science research, as demonstrated in the study of irrational religious orientations among Daoist and Buddhist believers. Grounded theory enables the development of theories that genuinely reflect respondents’ experiences, fostering a deeper understanding of the subject.

In this study, the researcher employed various grounded theory coding strategies, starting with open coding to extract key concepts, followed by principal axis coding to understand their interrelationships, and concluding with selective coding to build a comprehensive theoretical framework [27–30]. Semi-structured interviews, complemented by literature analysis, were pivotal in exploring the conceptual nuances of irrational religious orientations, enhancing the depth and applicability of the findings.

Overall, the researcher’s grounded theory approach, supported by relevant literature, illustrates its effectiveness in social sciences for examining complex phenomena like irrational religious orientations, confirming its vital role in current scholarly discourse. The research utilized a mix of online and offline interviews, guided by an outline of open-ended questions (see Table 1), ensuring comprehensive coverage of the research topic.

**Table 1:** Open-ended questions

Main part	Concrete content
1. Personal religious experience	Circumstances, frequency and motivation for visiting religious sites; use of religious objects or participation in religious activities; experience of using religion for solace in times of frustration
2. Observation of socio-religious phenomena	Perceptions of religious tourism among young groups; current trends in the commercialization of religion
3. Conceptual understanding of irrational religious orientations	Understanding of the term “irrational religious orientations”; causes and typical cases of irrational religious orientations; circumstances surrounding irrational religious orientations; existence of irrational tendencies in individuals
4. Reflections on Rational Faith	Qualities that should characterize rational religious beliefs; the problem of stability of beliefs; inclusiveness of different faith communities; and suggestions for fostering rational beliefs

This study aims to define irrational religious orientations, setting a foundation for future research. A diverse and inclusive sample was crucial, with 29 participants from varied backgrounds in terms of gender, age, occupation, and religious affiliation, ensuring broad representativeness (details in Table 2). The sample included a balance of genders (8 males, 21 females) and a wide age range (19–55 years), encompassing various professions like clerical staff, freelancers, and entrepreneurs, enriching the study with diverse professional insights.

The participants’ religious beliefs included only three beliefs, Taoism, Buddhism, and no religious beliefs, which comprehensively reflected their religious orientation. The study utilized a dual-mode interview method, i.e., offline interviews using a KDDI SR502 recorder in a quiet environment and online interviews via Tencent conferencing software to ensure effective communication and data collection. Each interview lasted between 30 and 50 min and was adjusted according to the comfort level of the participants to optimize data quality.

Ethically, the study upheld privacy and confidentiality standards, with voluntary participation emphasized, showcasing a commitment to ethical research practices.

**Table 2:** Basic information of participants

Participant No.	Genders	(A person’s) age	Careers	Religious background	Interview length/min
P1	male	52	SOE employeesr	Daoism	38
P2	male	24	Freelance	Daoism	32
P3	female	25	Freelance	Daoism	29
P4	female	22	Schoolchildren	Buddhist	31
P5	female	50	Yoga Sound Therapy Teacher	Buddhist	62
P6	female	21	Schoolchildren	Daoism	68
P7	female	21	Attorney	not have	19
P8	female	22	Attorney	not have	22
P9	female	22	Attorney	not have	18
P10	female	21	Attorney	not have	22
P11	female	22	Attorney	not have	19
P12	female	23	Schoolchildren	not have	33
P13	female	22	Schoolchildren	Buddhist	47
P14	female	22	Schoolchildren	Buddhist	58
P15	female	24	Anchor (TV)	Buddhist	23
P16	male	19	Schoolchildren	Daoism	63
P17	female	23	Schoolchildren	Buddhist	42
P18	male	25	Attorney	Buddhist	53
P19	female	22	staff member	Daoism	28
P20	female	25	Schoolchildren	Buddhist	32
P21	female	26	Schoolchildren	Daoism	27
P22	male	50	Engineer	Buddhist	18
P23	male	23	Schoolchildren	Daoism	19
P24	male	28	Schoolchildren	Buddhist	28
P25	female	50	SOE employees	not have	21
P27	female	55	Chinese Medicine Doctor	Daoism	49
P28	male	40	Entrepreneur	Daoism	53
P29	female	25	Venture Partner	Daoism	63

### Findings

This research, grounded in qualitative methodology, emphasizes the open coding process in analyzing interview transcripts, underscoring the crucial role of qualitative data analysis software like NVivo14 in categorizing data and conceptualizing themes [31–32]. Open coding involves a detailed dissection of data, here interview transcripts, to extract categories, properties, and hypotheses, demanding an in-depth understanding and identification of recurring patterns or themes [33]. This creative yet disciplined process requires an analytical mind capable of connecting disparate qualitative data.

NVivo14 is instrumental in breaking down data into manageable units, organizing and analyzing content to identify key themes and patterns [32]. This software minimizes category overlap, clarifying and distinguishing each theme, thereby enhancing the analysis’s accuracy and quality. The incorporation of digital tools like NVivo14 in research workflows not only speeds up the process but also ensures a thorough, nuanced examination of qualitative data. The specifics of open coding are presented in Table 3.

**Table 3:** Open codes

Coding	Descriptive	Data sources	Sample citation
C01 Blind Conformity	In religious practice and decision-making, individuals show a tendency to lack critical thinking and imitate the behavioral patterns of others without analysis.	P28	I am influenced by people around me in matters related to religion.
C02 Obsessive	It manifests itself in an over-attachment to the results of metaphysical predictions, leading to irrational and excessive actions by the individual in pursuit of their results.	P01	I will pay money to find a guru on the internet social media to predict the outcome of my marriage and career, and if the results are not satisfactory, I will feel that the guru is not good enough and will consider changing to another guru
C03 Coercion	It refers to the pressure exerted on others or oneself to conform to a particular desire or demand, ignoring the importance of voluntariness.	P06	I used to ask my friends or family members to go to a certain temple at a certain point in time to pray for blessings
C04 Wastefulness	It describes the behavior of individuals who engage in consumption beyond their actual needs in order to pursue external social status or display financial power, which leads to irrational use and waste of resources.	P16	I think spending more than you can afford on a pilgrimage to the Holy Land is an understandable behavior
C05 Blind Rendering	Refers to the dissemination of unverified information, opinions or statements by individuals without adequate verification of information or lack of forethought.	P03	I’d like to recommend the religious guru I just met to my friends and spread the word in my circle of friends
C06 Profit-making Tendency	Viewing religious beliefs and their associated activities as profit-making mechanisms, functionalizing them as a means of economic gain or commodifying them.	P02	If I had learned to tell fortunes or predictions, I would have made money by this method
C07 Compensation	Individuals seek psychological equilibrium by attempting to compensate for internal psychological deficiencies or dissolve feelings of personal loss through religious consumer behavior.	P13	Going to a temple or a Taoist temple makes me feel bad if I don’t pay for it
C08 Flaunting	Attempts to attract social attention and enhance one’s social status by magnifying and emphasizing one’s religious experience.	P06	I like to mention the temples and Taoist temples I’ve visited at social events and introduce them to the masters I’ve known
C09 Investing Tendency	Irrational investments in religious matters are made based on the motivation of expected high returns, not on religious beliefs per se.	P22	I think one’s luck gets better as one goes to the temple more often or spends more time and money on religion
C10 Flight from Reality	Individuals outsource responsibility for problem solving to external factors rather than actively exploring solution strategies.	P12	As long as real life is stressful, I want to go to the temple and stay there.
C11 Responsibility Evasion	Individuals tend to avoid taking responsibility and confronting realistic challenges in their behavioral patterns.	P24	“Between going to work and getting ahead, I’ll take the incense.” That’s a good reflection of where I’m at right now.
C12 Obedience to the Word	Individuals obey authority figures unconditionally and lack critical analysis of their instructions.	P17	I feel that if I don’t act on what the master says, everything will go wrong.
C13 Dogmatic Rigidity	Individuals adhere to established dogma and are unwilling to engage in rational self-reflection and knowledge renewal.	P19	Before you do anything, you’ll always get your fortune read or check your luck.
C14 Negative Coping	When faced with challenges, individuals tend to rely on religious strategies rather than positive solutions.	P14	In times of trouble, I find praying for divine blessings to be an effective approach
C15 Authoritative Dependence	When dependence leads to the loss of an individual’s ability to think and judge on his or her own, the individual becomes completely dependent on authoritative opinions and lacks critical thinking.	P18	I would like to meet a few more monks and Taoist priests in real life so that my future is very secure
C16 Double Standards	Apply inconsistent judging criteria for the same behavior or scenario based on individual identity or background.	P16	I feel uncomfortable when people comment on my favorite religion, but I have no such qualms when commenting on other religions.
C17 Social Isolation	Individuals tend to isolate themselves from external socialization with the same faith group, resulting in a restricted social circle.	P16	I can only find belonging in the Taoist/Buddhist community
C18 Emotional Dependence	Individuals are overly dependent on a particular religious object and have difficulty adapting to new environments if they lose it.	P05	I feel that life is painful and only by relying on the power of the gods can I feel better in my heart
C19 Persuasion	Persuading others to perform otherwise unwanted behaviors through delightful conversations and (sometimes untrue) promises.	P27	I’ll convince everyone around me to go to some temple with me to pray for blessings.
C20 Paranoia	Manifests as an abnormal, self-centered notion of superiority or unrealistic delusions.	P01	I feel like doing something related to religion at a certain time must be prioritized above all else

In grounded theory methodology, spindle coding follows open coding as a pivotal process [26]. Its primary role is to establish connections and relationships between pre-existing codes. This stage synthesizes initial codes into broader themes, revealing causal links, conditions, and contexts [34]. For instance, researchers might group codes into themes like “belief avoidance” or “belief dependence,” exploring their interplay within the studied phenomenon.

Selective coding, the final step in grounded theory [26, 35–36], integrates categories from spindle coding around a central or “core category“ [37]. This is done to form a cohesive theory around the core categories that effectively summarizes the major phenomena observed in the study [26, 34, 38]. This forms a unified theory reflecting the study’s main observations. For example, if “religious spiritual dependence” emerges as a core category, selective coding aligns all related categories to depict its representation in the data. This process culminates in a structured, coherent theoretical framework, as outlined in Table 4.

**Table 4:** list of grounded theory tertiary codes

Core scope	Main category	Subcategory	Scope
B01 Religious Spiritual Dependence	A01 Faith Evasion	C10 Reality Escape	Individuals outsource responsibility for problem solving to external factors rather than actively exploring solution strategies.
		C11 Responsibility Evasion	Individuals tend to avoid taking responsibility and confronting realistic challenges in their behavioral patterns.
	A02 Faith Dependence	C14 Negative Coping	When faced with challenges, individuals tend to rely on religious strategies rather than positive solutions.
		C18 Emotional Dependence	Individuals are overly dependent on a particular religious object and have difficulty adapting to new environments if they lose it.
	A03 Dissemination of faith	C19 Persuasion	Persuading others to perform otherwise unwanted behaviors through delightful conversations and (sometimes untrue) promises.
B02 Religious Instrumental Tendencies	A04 Extravagant Display	C04 Wastefulness	It describes the behavior of individuals who engage in consumption beyond their actual needs in order to pursue external social status or display financial power, which leads to irrational use and waste of resources.
		C08 Flaunting	Attempts to attract social attention and enhance one’s social status by magnifying and emphasizing one’s religious experience.
	A05 Profit-driven	C06 Profit-making Tendency	Viewing religious beliefs and their associated activities as profit-making mechanisms, functionalizing them as a means of economic gain or commodifying them.
		C09 Investing Tendency	Irrational investments in religious matters are made based on the motivation of expected high returns, not on religious beliefs per se.
	A06 Stubbornness and Narrow-mindedness	C02 Obsessive	It manifests itself in an over-attachment to the results of metaphysical predictions, leading to irrational and excessive actions by the individual in pursuit of their results.
		C20 Paranoia	Manifests as an abnormal, self-centered notion of superiority or unrealistic delusions.
		C13 Dogmatic Rigidity	Individuals adhere to established dogma and are unwilling to engage in rational self-reflection and knowledge renewal.
	A07 Social Avoidance	C17 Social isolation	Individuals tend to isolate themselves from external socialization with the same faith group, resulting in a restricted social circle.
B03 Religious Uniqueness Identity	A08 Critically Deficient Conformity	C01 Blind Conformity	In religious practice and decision-making, individuals show a tendency to lack critical thinking and imitate the behavioral patterns of others without analysis.
		C05 Blind Rendering	Refers to the dissemination of unverified information, opinions or statements by individuals without adequate verification of information or lack of forethought.
		C15 Authoritative Dependence	When dependence leads to the loss of an individual’s ability to think and judge on his or her own, the individual becomes completely dependent on authoritative opinions and lacks critical thinking.
	A09 Psychological Compensatory Beliefs	C07 Compensation	Individuals seek psychological equilibrium by attempting to compensate for internal psychological deficiencies or dissolve a sense of personal loss through religious consumer behavior.
	A10 Authoritative Dependence	C03 Coercion	It refers to the pressure exerted on others or oneself to conform to a particular desire or demand, ignoring the importance of voluntariness.
		C12 Obedience to the Word	Individuals obey authority figures unconditionally and lack critical analysis of their instructions.
	A11 Identity Judgemental Discrimination Blind Conformity	C16 Double Standards	Apply inconsistent judging criteria for the same behavior or scenario based on individual identity or background.

### Dimension construction process

The results of the semi-structured interviews on irrational religious orientations revealed three main dimensions of irrational religious orientations: B01 Religious Spiritual Dependence; B02 Religious Instrumental Tendency; and B03 Religious Uniqueness Identity.

#### Religious spiritual dependence

The three main dimensions are A01 Faith Escape, A02 Faith Dependence, and A03 Faith Dissemination, and the five subdimensions are C10 Reality Escape, C11 Responsibility Escape, C14 Negative Coping, C18 Emotional Dependence, and C19 Persuasion.

Religious-spiritual dependence reflects an individual’s excessive reliance on religious beliefs, which usually manifests itself in the form of avoidance of real-life difficulties and responsibilities, as well as the search for psychological comfort and a sense of social belonging [39]. In gaining a deeper understanding of the nature of this dependence, its multiple dimensions can be revealed by analyzing the three main categories - Faithful Evasion, Faithful Dependence, and Faithful Dissemination - and their related subcategories.

Faithful Evasion encompasses the subcategories of " C10 Reality Escape " and " C11 Responsibility Escape “. This concept describes the use of religious beliefs by individuals to escape real-life dilemmas and personal responsibilities, reflecting religious spirituality as a mechanism to avoid real-life challenges.

Seeking Solace in Faith is a fusion of the subcategories of “C14 Negative Coping” and “C18 Emotional Dependence”. It expresses the tendency of individuals to seek religion for emotional comfort and psychological support in the face of life’s challenges, rather than actively solving problems, and shows individuals’ reliance on the spirit of religion for psychological comfort and emotional support in the face of adversity.

Faith Dissemination, derived from the subcategory of “C19 Persuasion”, describes individuals who actively persuade others to accept their religious beliefs due to the need for spiritual dependence. This behavior may be due to the fact that the individual seeks to gain self-affirmation and psychological support by getting others to accept his or her beliefs.

Considering the relationship between these primary and secondary categories together, the complexity of religious spiritual dependence can be seen. Individuals may seek to cope with life’s stresses and challenges through faith escape, find emotional solace and psychological support through faith dependence, and enhance their own faith experience and increase their sense of social belonging through faith transmission. Together, these patterns of behavior constitute the structure of religious-spiritual dependence, reflecting how individuals respond to various psychological and social needs in their personal lives through religious belief.

#### Religious instrumental tendency

The 4 main categories are A04 Extravagant Display, A05 Profit-driven, A06 Stubbornness and Narrow-mindedness, and A07 Social Avoidance. the 8 subcategories are C04 Wastefulness, C08 Flaunting, C06 Profit-making tendency, C09 Investing tendency, C02 Obsessive, C20 Paranoia, C13 Dogmatic rigidity, C17 Social isolation.

Religious instrumental tendency is a state of mind that uses religious beliefs as a means to achieve personal ends, and this tendency shows diversity and complexity among different individuals. By analyzing in depth the four main categories - Ostentatious Display, Profit-Driven, Stubborn Narrow-mindedness, and Social Avoidance - and the sub-categories associated with them, we can understand the nature and manifestations of this tendency more fully.

The concept of “A04 Extravagant display”, formed by combining the subcategories of “C08 Flaunting” and “C04 Wastefulness”, describes the excessive and unnecessary consumption behaviors that individuals engage in in order to display their social status and wealth. Such behavior is not only intended to attract the attention and admiration of others, but also reflects a strong desire for social recognition and status in the context of religious instrumentalism.

“A05 Profit-driven” is a blend of “C06 Profit-making tendency” and “C06 Profit-making tendency”, and is characterized by the individual’s intense pursuit of monetary rewards. This mindset may drive individuals to seek profit in various investments and business activities, sometimes without regard for risk or ethics.

“A07 Social avoidance”, derived directly from the subcategory of “C17 Social isolation”, describes an individual’s tendency to avoid social interactions due to fear of interpersonal complexity or distrust of others. This avoidance behavior may be a defense mechanism, but in the long run it may lead to a deterioration of social skills and impoverishment of interpersonal relationships.

“A06 Stubbornness and narrow-mindedness” combines the subcategories of " C02 Obsessive,” " C20 Paranoia,” and " C13 Dogmatic rigidity,” highlighting a lack of openness and flexibility in an individual’s thinking and behavior. A lack of openness and flexibility in individual thought and behavior. Such attitudes are often associated with resistance to dissent and new information, and reflect an overly insistent and narrow perspective on religious ideas.

Considering these categories together, religious instrumental tendencies constitute a complex web of individual behaviors and mindsets. Through extravagant displays, individuals may seek social recognition and status; through the profit motive, they pursue material gain; through social avoidance, they avoid confronting the complexity of relationships; and through stubborn narrow-mindedness, they defend their beliefs and perspectives. Together, these patterns of behavior exemplify how individuals use religious beliefs to achieve personal ends, including the pursuit of material gain, social status, and avoidance of social interactions.

#### Religious unique attribute identity

The four main categories are A08 Critically Deficient Conformity, A09 Psychological Compensatory Beliefs, A10 Authoritative Dependence, and A11 Identity Judgemental Discrimination Blind Conformity. the seven subcategories are C01 Blind Conformity, C05 Blind Rendering, C15 Authoritative Dependence, C07 Compensation, C03 Coercion, C12 Obedience to the Word, and C16 Double Standards.

Religious uniqueness identity refers to specific mental attitudes and behavioral patterns exhibited by individuals in their religious practices and beliefs. These patterns typically include blind obedience to authority, compensation for psychological needs, and discrimination and prejudice in identity judgments. By analyzing the four main categories: critically deficient conformity, psychologically compensatory beliefs, authority attachment, and identity-judging discrimination, as well as the related subcategories, we can gain insight into the nature of religiously exclusive identity.

Uncritical Conformity combines the subcategories of Blind Conformity, Blind Rendering, and Authority Dependence to describe the nature of individuals’ religious practices. It describes an individual’s blind acceptance of authoritative opinions and collective beliefs in religious practice without individual critical thinking. This reflects the individual’s unconditional obedience to religious authority and collective views.

Psychological Compensation Faith retains the independence of the Compensation subcategory and emphasizes the use of religious practices to satisfy internal psychological needs, such as comfort, self-affirmation, or escape from stressful situations.

Compliance Pressure combines the subcategories of Compulsion and Obedience to emphasize the unconditional obedience of individuals to authority in religious contexts and the coercion of beliefs on others. It expresses the individual’s submissiveness and dependence on religious authority.

Identity Judgment Bias maintains the independence of the subcategory of “double standards”, which relates to the impartiality of judgments, and manifests itself in discrimination and prejudice against different identities or groups in religious beliefs and practices.

The relationship between these primary and secondary categories reveals the multiple dimensions of religious identity. Individuals may exhibit blind obedience to religious authority and collective viewpoints through critically deficient subordination; through psychologically compensatory beliefs that utilize religion to satisfy internal psychological needs; through authoritative dependence, which manifests as obedience to authority and coercion of beliefs about others; and through identity judgmental discrimination, whereby individuals may exhibit discrimination and prejudice against different identities or groups in their religious beliefs and practices. Together, these behavioral and attitudinal patterns constitute the complex structure of religious uniqueness identity, reflecting how individuals develop specific psychological attitudes and behavioral patterns in their religious beliefs and practices.

## Discussion

### Theoretical modeling of irrational religious orientations

**Fig. 1:**
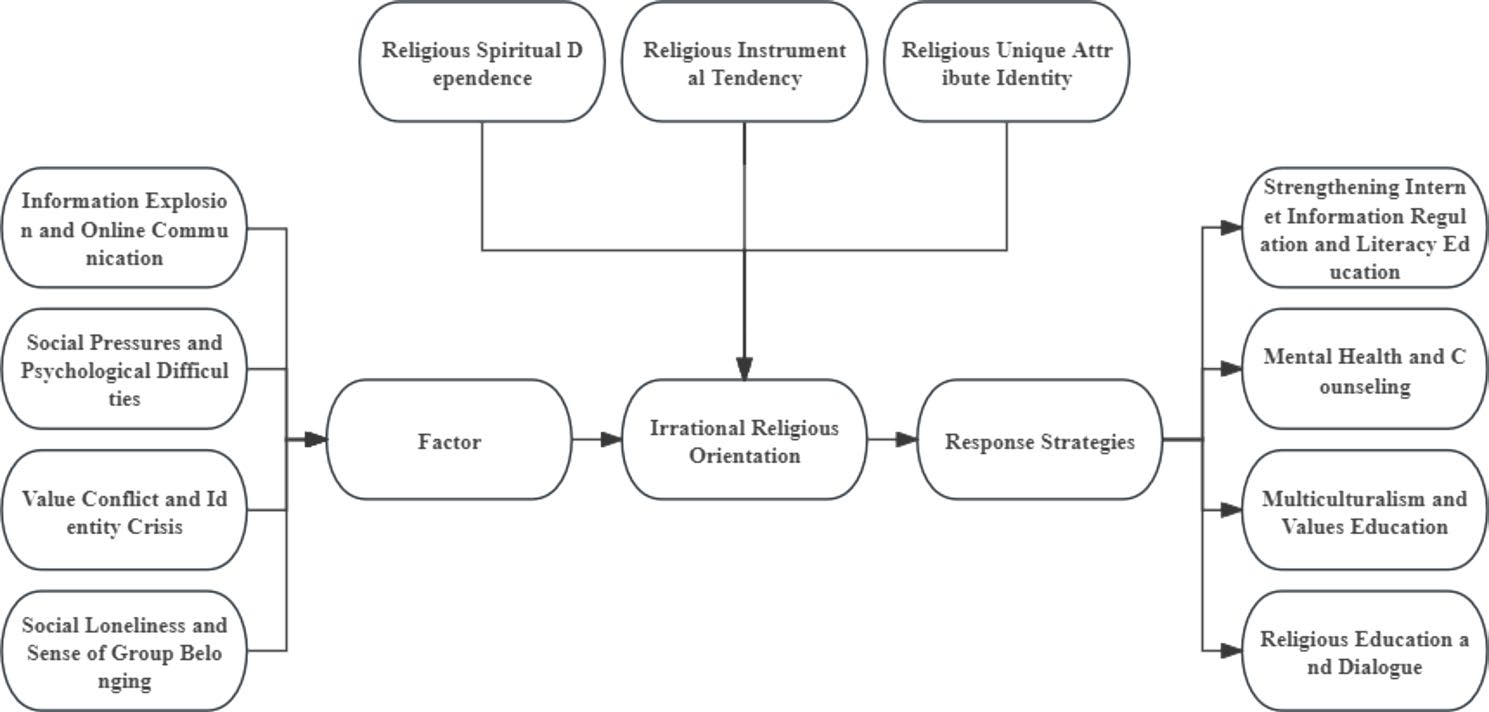
Conceptual Model of Irrational religious orientations

In constructing a theoretical model of irrational religious dispositions, a variety of complex psychological, social, and cultural factors are considered and how they interact to shape an individual’s religious behaviors and attitudes (as shown in Fig. 1). The model refines the key factors that shape irrational religious dispositions, explores their profound impact on individual mindsets and behaviors, and proposes a range of strategies aimed at mitigating or preventing these dispositions. In this framework, we can see how religious beliefs can mutate from a healthy spiritual support to an irrational form that can bring about psychological distress and social division. Next, we will explore in detail the factors that shape irrational religious orientations as well as specific measures to counter these tendencies.

### Factors shaping generation Z’s Irrational religious orientations

#### Information explosion and online communication

In the digital age, Generation Z is significantly impacted by the vast availability of online information, particularly in shaping their religious beliefs. This information overload often leads to cognitive stress and confusion, as they struggle to process and assimilate extensive religious content [40]. Consequently, decision-making becomes more challenging, and there’s a tendency towards superficial information processing [41]. The diversity of information, while offering broad perspectives, also poses risks. Extreme or irrational religious views online can mislead youth, impacting their value formation. Additionally, social media algorithms may reinforce existing beliefs, creating an ‘Echo Chamber Effect’ and hindering critical thinking [42–43].

Therefore, the information era presents both opportunities and challenges for Generation Z in forming religious concepts. The key concern is aiding them in effectively filtering and processing information to develop rational beliefs [44].

#### Social pressures and psychological difficulties

In the current social context, Generation Z faces considerable stressors, including academic, career, and social pressures, contributing to mental health issues like anxiety and depression [45]. To cope, many turn to religious beliefs for solace, sometimes adopting irrational religious ideas that offer simple solutions [45].

This reliance on irrational religious concepts can lead to avoidance behaviors, impacting long-term development and mental health [46]. Such avoidance may manifest as denial of real-life problems and a lack of constructive coping strategies.

This reliance on irrational religious concepts can lead to avoidance behaviors, impacting long-term development and mental health [47]. This avoidance behavior may manifest itself in ignoring or denying real-life problems, as well as a lack of positive coping attitudes in the face of difficulties.

Furthermore, excessive reliance on these beliefs in decision-making can impair rational thinking, leading to potentially harmful choices in education, career, health, and relationships [48–49].This can also result in a disconnect from family, friends, and society, potentially leading to social isolation and increased psychological distress [41, 50].

In summary, Generation Z’s turn to irrational religious beliefs as a response to societal and psychological pressures not only affects their mental health and development but also influences their social relationships and life choices. Understanding and addressing these tendencies is vital for their overall well-being.

#### Value conflict and identity crisis

In the context of globalization and the digital era, Generation Z navigates complex challenges in shaping their identity and values [51]. This generation actively seeks to establish unique identities, often blending various cultures, values, and lifestyles beyond traditional or ethnic boundaries. Balancing cultural conflicts and integration, they grapple with tradition versus modernity and local versus global influences.

Many in Generation Z question traditional religions and cultural values, gravitating towards non-mainstream or emerging religious beliefs as a form of spiritual solace and a means to express individuality and dissent [52–53]. This exploration is also driven by their need for a sense of community and belonging. They often turn to virtual communities, which offer a platform for aligning with specific religious concepts or lifestyles, providing a new avenue for identity formation and belonging [54–55].

Overall, Generation Z’s journey in forming personal identities and values is influenced by a mix of cultural diversity, individualization, and community belonging. This journey often includes an attraction to non-mainstream religious beliefs, highlighting the complexity of their search for identity and belonging.

#### Social loneliness and sense of group belonging

The rise of social networks has had a profound dual impact on Generation Z’s social habits and religious perspectives [56]. Social media, while facilitating connectivity, often lacks depth and authenticity, leading to decreased real-life socialization and potential social isolation [57–58]. The culture of online comparison can undermine self-worth, exacerbating feelings of loneliness and dissatisfaction. Additionally, overreliance on virtual communication may impair real-life social skills, hindering the formation of meaningful relationships [59].

In response, religious groups are becoming increasingly appealing to Generation Z for offering community and a sense of belonging [50, 60]. The shared beliefs and community activities within these groups can mitigate feelings of isolation and promote social engagement. However, in their search for belonging and meaning, Gen Z may also be drawn to irrational religious beliefs that provide simple answers to complex life questions.

In summary, social media’s influence and the resulting social isolation may prompt Gen Z to seek belonging in religious communities, while simultaneously increasing their susceptibility to irrational religious orientations. This underscores the complexities of Gen Z’s pursuit of social connection, psychological solace, and identity formation.

### Response to Generation Z’s Irrational religious orientations

#### Strengthening internet information regulation and literacy education

Enhancing internet information regulation and literacy education is vital in assisting Generation Z to discern and resist irrational religious orientations, fostering the development of sound religious concepts and values.

Effective online information regulation involves scrutinizing and filtering religious content to prevent the spread of misinformation and extreme ideas [61–62]. This includes restricting misleading content and ensuring online platforms are transparent and accountable, flagging or removing content promoting harmful religious ideologies [63].

Literacy education should focus on equipping Gen Z with skills to critically analyze internet content, particularly religious information. This involves teaching them to identify credible sources, understand the intentions behind information, and evaluate online content from various perspectives [64–65].Media literacy education, crucial for safe and responsible use of social media and online platforms, should be integrated into school curricula and broader societal education [66]. A comprehensive approach requires multifaceted education and guidance, extending beyond formal school settings to families, communities, and online platforms. Promoting content that disseminates healthy religious concepts and recognizing individual differences in information processing and critical thinking are also key. Personalized support should be offered based on individual needs [63–66]. These strategies will help Generation Z to develop healthy and rational beliefs and values.

#### Mental health and counseling

Mental health education and counseling are pivotal for assisting Generation Z in managing psychological stress and diminishing their reliance on irrational religious beliefs. Firstly, enhancing mental health awareness is crucial. It involves educating young people about recognizing and understanding common psychological issues, like anxiety and depression, which are fundamental for mental well-being [67].

Developing coping and emotional self-regulation skills is also essential. This approach teaches Generation Z effective strategies for handling life’s pressures and emotional challenges, enabling them to respond positively to mood swings and frustrations [68].

Professional psychological counseling plays a significant role, providing emotional support and specialized assistance, especially in addressing personal problems and stress. Tailored counseling services offer individualized support, helping young people discover personal coping strategies [69–70].

Implementing these strategies in schools and communities is equally important. Integrating mental health education into curriculums and providing accessible community resources, like hotlines and workshops, broadens support. Training parents and teachers enhances their ability to understand and meet the psychological needs of youth [71].

A comprehensive approach includes a multi-channel mental health support system, combining resources from educational institutions, families, communities, and professional organizations. This system should foster open discussions about mental health to dismantle taboos and offer specialized support for those with specific psychological needs [72].

Through these initiatives, Generation Z can more effectively manage psychological stress, enhancing their mental health and reducing dependency on irrational religious practices, thereby promoting their overall well-being and healthy development.

#### Multiculturalism and values education

Multiculturalism and values education are essential in addressing the irrational religious inclinations of Generation Z [73]. This form of education fosters an appreciation and respect for diverse cultural backgrounds, beliefs, and traditions, crucial for cultivating a broad-minded perspective among young people [74]. It enhances cultural awareness, sensitivity, and the ability to respect and value equality, while also sharpening critical thinking skills [75].

The role of public media is significant in promoting pluralistic and inclusive narratives. By offering varied perspectives, including those of minority and marginalized groups, media can contribute to a balanced understanding while steering clear of extreme or radical viewpoints.

Implementing systematic multicultural and values education programs involves integrating these themes into school curricula and leveraging the influence of public media. Encouraging active participation in social and cultural activities enables young people to engage with diverse groups, fostering practical experiences in multiculturalism and values. Opportunities for volunteerism, community involvement, and cultural experiences further reinforce these concepts [73–75].

These strategies are pivotal in helping Generation Z develop a rational worldview, mitigate the allure of irrational religious beliefs, and grow into open, tolerant, and understanding members of a multicultural society.

#### Religious education and dialogue

Religious education is key in enhancing young people’s comprehension and appreciation of various religions [76]. It delves into the history, core beliefs, and practices of different faiths, emphasizing the spectrum and intricacies of religious beliefs. This education is instrumental in helping Generation Z understand diverse religious perspectives, recognize similarities and differences among them, and discern between rational religious concepts and extremist ideas [77–78].

Public lectures and seminars featuring religious experts can foster dialogue and rational discussions, enabling students to articulate and respect diverse viewpoints [79–80]. Inter-religious exchange activities further promote mutual understanding and respect across different faiths.

Critical thinking is a cornerstone of religious education, equipping young people to analyze and critically evaluate religious information, identify prejudices, and base their understandings on facts and logic [81–82]. It encourages them to develop their own religious views, rather than conforming to others’ beliefs uncritically.

Effective strategies for promoting religious understanding include offering religious education and dialogue through schools, communities, religious institutions, and public media. Emphasizing inclusivity and respect for both believers and non-believers in all forms of religious education and dialogue is essential. These measures are designed to help Generation Z develop a well-rounded worldview, understand the role of religion in personal and societal contexts, and become more open, inclusive, and rational members of society [76, 80–82].

## Conclusion

### Main findings

This research delves into the irrational religious orientations of Generation Z in China, uncovering their complexity and multidimensionality. The study identifies key aspects:

**a. Religious Spiritual Dependence**: This includes faith avoidance, dependence, and propagation, highlighting how individuals excessively rely on religious beliefs for psychological comfort and social belonging when facing real-life challenges.

**b. Religious Instrumental Tendency**: Some individuals use religion as a means to achieve personal goals, such as gaining social status or material benefits.

**c. Religious Uniqueness Identity**: This reflects specific attitudes and behaviors in religious practices among Generation Z, characterized by a lack of critical thinking and using religion to fulfill psychological needs.

The formation of these tendencies is influenced by factors such as information overload and Internet communication, leading to cognitive challenges and susceptibility to misinformation, particularly in developing religious ideas. Social pressures, academic and professional development challenges, value conflicts, identity crises, social isolation, and the need for group belonging also contribute significantly.

The study suggests multifaceted strategies to address these tendencies:

**Promotion of Multiculturalism and Values Education**: Through education and public media, fostering respect, equality, and critical thinking.

**Strengthening Online Information Regulation and Literacy**: Aiding Gen Z in discerning information and developing rational religious concepts.

**Mental Health Awareness and Counseling**: Supporting Gen Z in managing psychological stress and reducing dependence on irrational religious beliefs.

**Encouragement of Social and Cultural Activities**: Enhancing communication and understanding among diverse groups, promoting openness and inclusivity.

These findings and strategies provide valuable insights into Generation Z’s irrational religiosity and propose practical approaches for support and guidance. Implementing these strategies is key to understanding their psychological and behavioral patterns in religious beliefs, crucial for their well-being and healthy development.

### Theoretical and practical implications

This study offers a comprehensive exploration of irrational religious orientations in China’s Generation Z, shedding light on their complex motivations and multidimensional nature. It examines how religious spiritual dependence, instrumental tendency, and exclusive identity interplay with personal behaviors, providing valuable theoretical insights into this complex phenomenon.

The research underscores the significance of cultural context in understanding religious orientations. Investigating these tendencies across various cultural and social environments can yield more nuanced understanding, highlighting the impact of cultural factors on the development and manifestation of these inclinations.

For policymakers, the study’s findings offer crucial guidance. It suggests the need for educational policies, public communication strategies, and social interventions tailored to address irrational religious orientations among Generation Z. These strategies aim to foster social cohesion and support the healthy development of young people.

Educators and mental health professionals can leverage these insights to better assist Generation Z. Multicultural education, cyber literacy, and mental health counseling emerge as key tools for guiding young people towards healthier, more rational religious attitudes and helping them navigate the psychological and social challenges associated with these tendencies.

In summary, this study not only enriches the theoretical understanding of irrational religious orientations but also provides practical strategies for addressing these issues, particularly focusing on Generation Z in China. Its implications are vital for enhancing societal well-being and fostering healthy development.

### Limitation

There are still some limitations of this paper, which are as follows:

#### Sample diversity

The sample in this study may not be fully representative of the broader Generation Z population in different parts of China, which may affect the generalizability of the results. The sample is limited to participants from predominantly urban areas, which may not reflect the religious orientation of participants from rural areas.

#### Methodological limitations

While grounded theory provides reliable qualitative insights, the interpretation of the data may be subjective and influenced by the researcher’s viewpoint. This may affect the neutrality and replicability of the study.

#### Cross-sectional nature

The study design is cross-sectional, which limits the ability to capture changes in religious orientation over time or to infer causal relationships between observed factors and religious orientation.

#### Reliance on self-reported data

The study relies heavily on self-reported data obtained through interviews, which are susceptible to biases such as social desirability or recall bias. Participants may present themselves in ways that they find socially acceptable rather than reflecting their true religious orientation.

#### Digital influences

Given the study’s focus on the digital influences of Generation Z, the study may overemphasize the impact of digital media on religious orientation without sufficiently considering other important influences such as family, education, and personal experiences.

### Recommendations for future research

To further enrich the understanding and theoretical framework of irrational religious orientations among China’s Generation Z, this study suggests employing innovative qualitative research methodologies such as Online Photovoice (OPV), Online Interpretative Phenomenological Analysis (OIPA), and Community-Based Participatory Research (CBPR) [83]. These methodologies are crucial for capturing the personal experiences and perceptions of individuals authentically and vividly, delving deeper into their thoughts, feelings, images, and behaviors.

Utilizing OPV and OIPA can provide valuable insights into the irrational religious orientations and their psycho-sociological implications. By applying OPV and CBPR, researchers can gain a deeper understanding of Generation Z’s approach to religious and spiritual concepts. Furthermore, examining religious and spiritual facilitators and barriers for Chinese people through the lens of OPV and OIPA, while collaborating with them from a CBPR perspective, is essential [84].

OPV, as one of the most recent and effective innovative qualitative research methods, offers a unique opportunity for participants to express their own experiences with minimal manipulation compared to traditional quantitative methods. Early adopters of OPV, such as Tanhan and Strack, have operationalized and explained it step by step, demonstrating its effectiveness in capturing authentic participant experiences [85].

Future researchers are encouraged to conduct qualitative or mixed-method studies to explore the potential of OPV. Educators and trainers can also use OPV for experiential activities to enhance group and organizational synergy. OPV and OIPA provide straightforward and comprehensive approaches to data analysis, resulting in meaningful and comprehensive insights [86].This approach is not only about expanding the understanding of irrational orientations but also about exploring the multi-dimensional aspects of religious spiritual dependence, instrumental tendency, and exclusive identity.

This comprehensive exploration provides critical insights into the interplay between religious beliefs and personal behaviors, offering new perspectives that contribute to a more nuanced understanding of this complex phenomenon. The study highlights the importance of conducting similar research in different cultural contexts, as cultural factors significantly influence the formation and manifestation of religious orientations. By analyzing these orientations in varied settings, researchers can obtain more comprehensive insights that enhance our understanding globally.

The findings from this study serve as important guidance for policymakers, suggesting the need for more effective educational policies, public communication strategies, and social interventions. These can be specifically targeted to address the challenges posed by irrational religious orientations and promote social cohesion and the healthy development of young people. Additionally, educators and mental health professionals can utilize these findings to better understand and support Generation Z. Through targeted interventions such as multicultural education, cyber literacy, and mental health counseling, professionals can guide young people to develop healthier and more rational religious attitudes, assisting them in navigating the psychological and social challenges associated with irrational religious orientations.

Overall, the integration of these innovative methodologies and the in-depth analysis provided by this study significantly contribute to the theoretical and practical understanding of irrational religious orientations. This is particularly significant in enhancing the well-being and promoting the healthy development of society, especially within the context of Generation Z in China.

## Data Availability

No datasets were generated or analysed during the current study.
